# Disability training for healthcare workers in Uganda: qualitative findings from the pilot test

**DOI:** 10.1186/s12909-025-07330-4

**Published:** 2025-05-23

**Authors:** Tracey Smythe, Andrew Sentoogo Ssemata, Abdmagidu Menya, Femke Bannink Mbazzi, Hannah Kuper

**Affiliations:** 1 International Centre for Evidence in Disability, London School of Hygiene & Tropical Medicine, Keppel St, London, WC1E 7HT UK; 2 Division of Physiotherapy, Department of Health and Rehabilitation Sciences, Stellenbosch University, Cape Town, South Africa; 3 Medical Research Council, Uganda Virus Research Institute and London School of Hygiene & Tropical Medicine Uganda Research Unit, Entebbe, Uganda; 4 Department of Global Health and Development, London School of Hygiene & Tropical Medicine, London, UK

**Keywords:** Disability, Training, Healthcare worker, Education, Low- and middle-income, Development, Uganda, MRC framework, Kirkpatrick’s model

## Abstract

**Background:**

People with disabilities experience barriers to healthcare, resulting in poorer health outcomes. There is limited disability training for healthcare workers globally. A disability training was co-developed with people with disabilities and healthcare workers and pilot-tested in Uganda.

**Objective:**

To use qualitative methods to understand co-learning experiences, identify strengths and areas for improvement, and to explore a disability training’s effect on practices in Uganda.

**Methods:**

We conducted a two-day Train-the-Trainer programme in September 2023 with ten trainers (5 people with disabilities, 5 healthcare workers). The trainers then delivered two one-day disability training programmes to 27 healthcare workers. Data on the perceptions and experience of the trainings were collected through focus group discussions with trainers and in-depth interviews with trainers and participants. We used an inductive approach for analysis and Kirkpatrick’s Four-Level Training Evaluation Model to assess reactions, learning, behaviour changes, and results.

**Results:**

The trainers valued the emphasis on practical application and the collaborative approach used during the sessions. Trainers with disabilities expressed increased confidence and ability to advocate for inclusive healthcare practices. Healthcare workers reported that the training was engaging and relevant to their roles. Three months post-training, healthcare workers reported improved attitudes and skills toward providing care for people with disabilities. Challenges in applying new practices included limitations in facility accommodations and accessibility. Further support and training were requested.

**Conclusion:**

The co-designed disability training programme can enhance healthcare workers’ skills and interactions with patients with disabilities. Policy support is important for the implementation of disability training at scale.

**Supplementary Information:**

The online version contains supplementary material available at 10.1186/s12909-025-07330-4.

## Introduction

There are 1.3 billion people with disabilities globally. On average, people with disabilities are more likely to experience poor health compared to the general population [1], and they have a two-fold higher mortality rate than people without disabilities [2]. Health systems need to be strengthened to address these inequities and to realise the right to health for people with disabilities [3]. A key concern is that many healthcare workers lack the necessary knowledge, skills, and attitudes towards disability [4]. Integrating disability education into healthcare training programmes is therefore vital to equip healthcare workers with the tools and resources to deliver respectful, culturally sensitive care [5], and is recommended by the WHO [6]. However, there are limited disability training programmes available globally [7], including in Uganda. In Uganda, disability is often still viewed through a medical or charity-based model, with limited integration of inclusive approaches within health system planning and delivery, especially in rural areas [8].

Training on disability can enhance healthcare workers’ knowledge, attitudes, and skills throughout their careers. For example, medical students in the U.S. improved their competencies in treating patients with disabilities after disability training was integrated into their curriculum [9]. Simulations helped students improve their communication and competency in Sweden, leading to better preparedness for interacting with patients with communication disabilities [10]. In Rwanda, a training programme that focused on childhood disability improved clinical decision-making skills among healthcare workers through immediate feedback and practical exercises [11]. Programmes that included people with disabilities as teachers also found that these interactions improved participants’ comfort and attitudes toward disability [12, 13].

Despite global recognition of the need for well-trained healthcare workers to improve health outcomes, there is a lack of standardised disability training programmes [7], especially in LMICs where the majority of people with disabilities live [6]. To address this gap, we co-designed a disability training programme with people with disabilities and healthcare workers [14]. The training focuses on key themes such as understanding disability rights, effective communication, informed consent, and disability-inclusive practices. The one-day disability training programme is delivered jointly by a person with a disability and a healthcare worker. The materials are developed as modules, and trainers incorporate interactive workshops, case studies, and peer learning. The disability training programme aims to enhance the knowledge, skills, and attitudes of participants towards providing disability-inclusive care, and promote respectful and effective interactions with patients with disabilities. The training emphasises cultural sensitivity and practical skills and engages people with disabilities as trainers to build mutual understanding and improve the quality of care provided.

This study aims to explore the perspectives and experiences of trainers and participants in a pilot study of the disability training in Uganda. We used qualitative methods to understand learning experiences, identify strengths and areas for improvement and potential effects of the training on practices in Uganda. A future study will evaluate the impact of the training material at scale.

## Methods

People with disabilities and healthcare workers co-developed the disability training programme in Uganda [14]. We used the Medical Research Council (MRC) approach, a systematic and evidence-based framework, to inform the development of the training programme [15].

### Study design

We conducted a pilot study in Uganda (26th Sept 2023–3rd March 2024) in Entebbe and rural areas of Luuka district using qualitative approaches. The study aimed to understand the perspectives and experiences of trainers who learned to deliver the disability training, and to identify strengths and areas for improvement within the materials. We also sought to identify the potential impact of the training on participants’ practices three months post-training. Additionally, we sought to gain an in-depth understanding of areas in the training to refine and enhance, and to identify the short-term effects of the programme. We adhered to the Consolidated Criteria for Reporting Qualitative Research (COREQ) checklist [16].

### Study setting

Luuka is a rural district in Eastern Uganda and has an estimated disability prevalence of 12% among those aged 5 and older. The district is served by 43 primary health facilities, which cater to an estimated population of 238,000 residents. The healthcare system in Luuka is structured with village health teams (VHTs) and three levels of health centres: Health Centre II (HC II), Health Centre III (HC III), and Health Centre IV (HC IV). Health care in the district is mainly provided by public and private not-for-profit healthcare service providers, community healthcare workers and traditional healers.

### Disability training intervention

A two-day Train-the-Trainer (TTT) course took place from September 26th to 28th, 2023, with ten trainers in Entebbe. The Trainers were identified from Bukanga subcounty in Luuka District and included five people with disabilities (a teacher, farmer, plumber, community facilitator, and social worker) and five healthcare workers (two clinical officers, a nurse, midwife, and laboratory assistant). Trainers with disabilities included those with visual impairment, physical impairment and albinism. Trainers were purposively selected from within the district as they understand the context. They were identified through local community networks and recommendations from district health officials. The TTT course involved trainers who were healthcare workers and people with disabilities learning together, focusing on effective training techniques, adult learning principles, participatory methods, and an introduction to the training materials. The trainers practiced delivering parts of the course in pairs, one person with a disability and one healthcare worker. The trainers then conducted and led a one-day disability training programme in Luuka district with two groups of healthcare workers of various cadres (*n* = 16 and *n* = 27) on October 4th and 5th, 2023. Participants were selected from 5 health facilities in Bukanga sub-country by local health authorities.

### Participant selection

Participants for the qualitative research were selected using a purposive sampling method to ensure diverse perspectives. They were approached face-to-face during training sessions and with a phone call 3 months after the training. The study included a total of 27 participants (17 healthcare workers and 10 trainers). No participants declined to participate.

### Data collection

Data were collected through two focus group discussions (FGD) and 27 in-depth interviews. The FGD were conducted in English and Lusoga by AM and EN, and were held separately with trainers immediately after the TTT: one group with trainers with disabilities and the other with healthcare worker trainers. The FGD were approximately 90 min and open-ended questions (Supplementary file 1) elicited detailed responses about the trainers’ perspectives, experiences, and learning. Audio recordings were transcribed verbatim, and translations were checked for accuracy.

Three months after the pilot disability training, in-depth interviews were conducted with 17 healthcare workers who had participated in the one-day disability training, as well as the 10 trainers. The interviews were held at participants’ workplaces or chosen locations. All discussions were audio-recorded with informed consent. These were complemented by field notes taken during and after sessions. No repeat interviews were conducted with participants. Audio recordings were transcribed verbatim, and those in Lusoga were translated into English. Translations were checked for accuracy. Interviews typically lasted 60 min and data saturation was achieved and discussed during data analysis. Transcripts were not returned to participants for comment or verification.

### Research team and reflexivity

The interviews and FGD were conducted by social science researchers (ASS, AM, EN) with extensive experience in disability studies and who had undergone specialised training in qualitative research methods. A relationship with the participants was established prior to the study, and participants were informed about the researchers’ professional backgrounds and the purpose of the study. The researcher disclosed their interest in disability training and aimed to maintain objectivity, although personal biases and assumptions regarding the importance of inclusive training methods were acknowledged.

### Data analysis

**Fig. 1:**
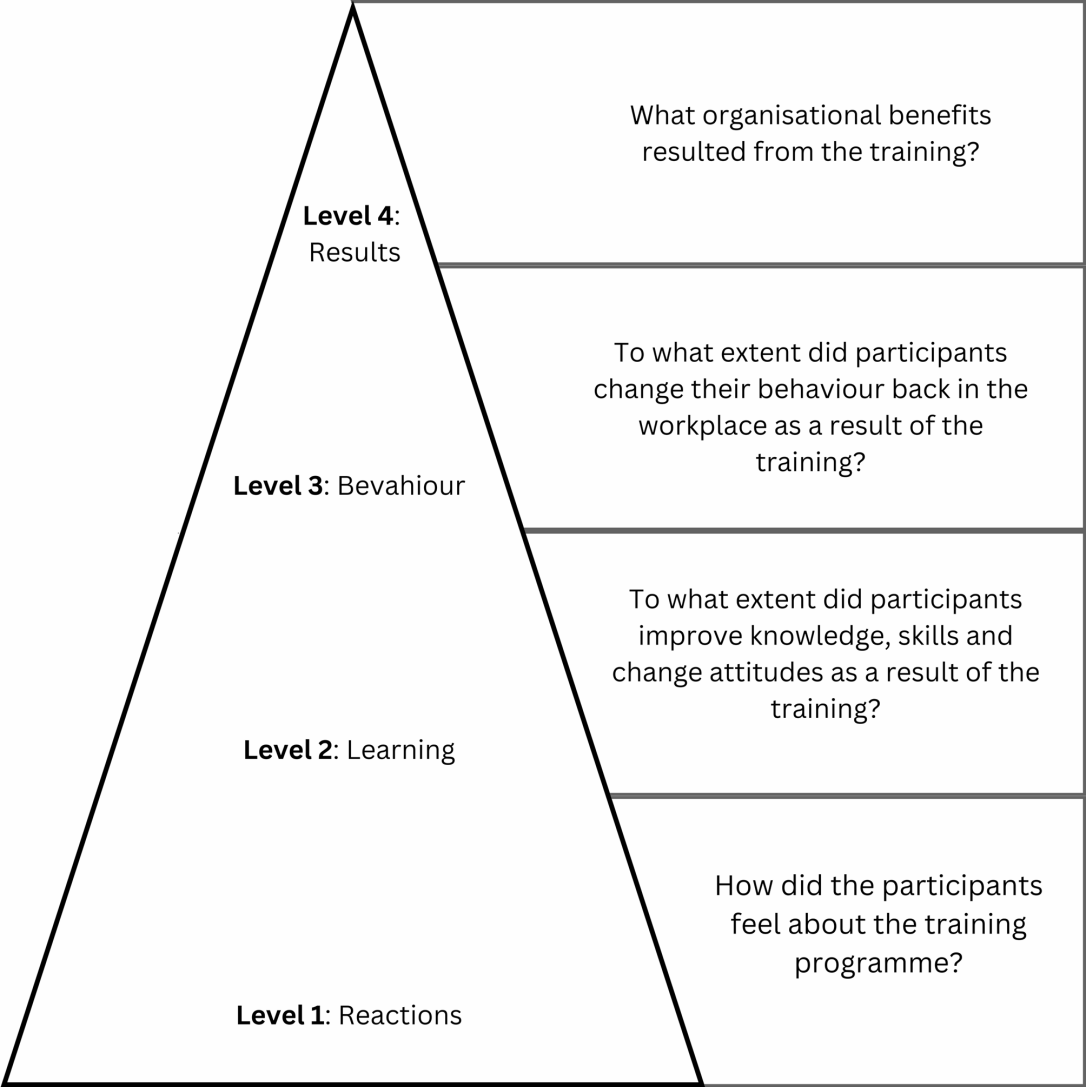
Kirkpatrick’s four-level training evaluation model

An analysis plan was developed collaboratively by three researchers (TS, ASS, AM) and was reviewed collectively to ensure consistency and comprehensiveness. We followed an inductive approach to allow themes to emerge directly from the data collected through interviews and FGDs. Initial coding was independently conducted by two researchers (TS and AM), followed by collaborative coding by all three (TS, AM, ASS). NVivo 14 was used for data management. We then applied thematic analysis to identify key patterns, which were subsequently mapped onto Kirkpatrick’s Four-Level Training Evaluation Model to structure our findings (Fig. 1.), focusing on participants’ reactions, the knowledge they gained, changes in their behaviour, and the overall results of the training. This two-step approach allowed us to remain grounded in participants’ perspectives while also providing a structured lens to interpret findings.

### Data trustworthiness

Initial and subsequent coding was performed by three researchers (AM, ASS, TS), to allow for comparison and reduce individual bias. Discrepancies in coding were resolved through discussion and consensus among the three researchers to ensure a reliable interpretation of the data. The analysis plan and coding process were reviewed by all three researchers to maintain consistency and rigor throughout the study. Reflexivity was maintained by acknowledging the researchers’ potential biases and their impact on the analysis.

To enhance credibility, we used investigator triangulation, with multiple researchers independently analysing the data and discussing interpretations to minimise bias. We addressed transferability by clearly describing the study context, participant characteristics, and setting, allowing others to assess the applicability of findings to similar contexts. To promote dependability, we followed a structured analysis plan and documented key decisions throughout the research process. Confirmability was supported by maintaining an audit trail and ensuring that all findings were grounded in the data through collaborative coding and regular team reflection.

### Ethical considerations

Ethical approval was granted by the Uganda Virus Research Institute Research and Ethics Committee (UVRI REC Ref: GC/127/904) and the London School of Hygiene & Tropical Medicine Observational Research Ethics Committee (LSHTM Ref 28327-1). Research clearance was obtained from Uganda’s National Council for Science and Technology (UNCST Ref: SS1348ES). Written informed consent was obtained from all participants. The study was conducted in accordance with the Declaration of Helsinki. To maintain data confidentiality, all transcripts were anonymised by removing identifying information from transcripts. The data were securely stored in password-protected files, accessible only to the research team. Participants were informed about confidentiality protocols and their rights regarding data usage.

## Results

Healthcare workers reported being highly satisfied with the disability training and valued the firsthand experiences shared by trainers with disabilities. Trainers accepted the paired delivery model, in which one trainer was a person with a disability and the other a health worker, and appreciated how the model combined personal experiences with professional insights. This approach was believed to enhance credibility and participant engagement. After 3 months, healthcare workers reported more positive attitudes toward people with disabilities and improved workplace policies, such as through the provision of reasonable accommodations and prioritised appointment times. However, some healthcare workers faced challenges due to limited institutional support and resources at their facilities. They also requested ongoing mentorship.

### Level 1: reaction– how did participants feel about the training programme

The TTT was well-received and considered engaging by the trainers who were healthcare workers. Trainers expressed readiness to pass on their knowledge. One trainer stated,

“*We are change agents! Now that we have been empowered*,* I anticipate that we will empower our colleagues*,* and that this will be passed on to the other teams*” (Trainer, HCW, female).

All trainers recognised that pairing a trainer with a disability and a trainer who is a health worker fostered collaboration and deeper understanding. This integration was seen as crucial to addressing ongoing discrimination within healthcare services and enhancing mutual respect. One of the trainers noted,

“*Bringing us together allows us to collaborate and helps the healthcare workers understand many facts about the disability… it was fantastic because healthcare workers and people with disabilities sat together to understand their requirements*” (Trainer, HCW, male).

While the use of paired trainers was well-received, delivering participatory training posed challenges, particularly as trainers were accustomed to traditional didactic methods. This discrepancy in training styles led to difficulties in understanding and implementing participatory activities and required ongoing support through demonstration, practice and mentorship to assist in the transition.

Participants of the disability training responded positively and appreciated the use of diverse methods such as demonstrations, group activities, case studies and role play to assist their learning and understanding. These approaches encouraged active engagement, with some healthcare workers noting,

“*We all needed to fully participate in the training*,* that was good*.” (HCW, male).

“*If someone with a disability tells you to do a certain thing*,* you truly understand what it means*” (HCW, female).

The involvement of trainers with disabilities helped address fears and misconceptions, allowing participants to connect more deeply with the material. Engaging in real-life experiences made the lessons more memorable, as other participants remarked,

“*It made us reflect on the things we used to do to people with disabilities*,* and you cannot make those mistakes again*” (HCW, female).

“*You took your time to interpret and explain for us to understand*,* therefore I am grateful*” (HCW, female).

The training created an inclusive environment where healthcare workers felt supported and understood. Participants also identified areas for improvement. Many felt that the training was too condensed into a single day, making it difficult to absorb all the information. Participants also highlighted the need for longer, repeated sessions to ensure lasting, effective change in healthcare practices. There was a desire for more in-depth exploration of certain topics, such as providing healthcare for people with sensory impairments, which may not be visible.

### Level 2: learning– improvement in knowledge, skills and attitudes

The TTT enhanced the trainers’ knowledge about disability as well as participatory learning techniques. Trainers who were healthcare workers valued learning from a trainer with a disability, recognising gaps in their knowledge and appreciating the new perspectives gained. Trainers highlighted essential skills such as learning to communicate, listening effectively, and passing information to others, which they could now better apply in both small and large group training settings.

The TTT bolstered their confidence and equipped trainers with the knowledge to advocate for inclusive healthcare and challenge prevailing stigma. It also shifted the perceptions of the trainers with disabilities regarding their own and others’ abilities and potential, and addressed misconceptions about the life expectancy and societal value of people with disabilities. One trainer reflected,

“*I assumed that people with disabilities were only here for a short time… the training has changed my perception*” (Trainer, with a disability, female).

Another trainer noted,

“*I had the impression that no one knew or valued me…you are disabled and your opinions are also disabled…it has given me great courage*,* which I will use to support and lead other people with disabilities*.” (Trainer, with a disability, male).

The pair of trainers learned to recognise and challenge their own biases during the delivery of the disability training. Healthcare workers developed a deeper understanding of inclusive practices, while trainers with disabilities gained confidence in their role as essential contributors to shape inclusive healthcare practices.

Participants of the one day disability training reported improvements in their understanding of disability, and noted that they now view people with disabilities as more autonomous and with value. One participant reflected,

“*Before the training*,* I was among those people who think that if a person has a disability*,* they are hopeless and a loss… However*,* that day of training was enough for me to change my perception and thinking*” (HCW, _female).

This change shows a move from viewing people with disabilities as a burden to a more respectful approach, acknowledging the individuality and autonomy of people with disabilities.

*“Since the training*,* I learnt that people with disabilities are also human*,* and when they come to the facility*,* I try all my best to provide them with the services they need… I used to call them by their disabilities. I used to see them as useless”* (HCW, male).

The training provided information on different forms of communication and participants also gained practical skills, such as using basic sign language. Healthcare workers gained new skills (e.g. assessing people with disabilities and strategies for effective referrals) which reshaped their understanding and reduced fear in treating patients with disabilities. One participant noted,

“*I learned how to communicate and interact with someone who is deaf*,* at least I can greet them which was not the case before.*” HCW, female)

The findings suggest that the disability training effectively introduces healthcare workers to both the social and human rights models of disability. The social model focuses on societal barriers as the cause of disability [17], while the human rights model emphasises dignity, autonomy, and equality for people with disabilities [18]. Both models challenge the medicalised view of disability and highlight the importance of societal change. Healthcare workers showed an understanding of how societal barriers contribute to the limited access to healthcare for people with disabilities in Uganda, as well as recognising their shared humanity and the need for inclusive care.

### Level 3: behaviour– changes in behaviour

The disability training programme was viewed by trainers with disabilities to improve the attitudes and behaviours of healthcare workers towards patients with disabilities. Trainers with disabilities noted that prior to the training, many healthcare workers were unfamiliar with the specific needs and rights of people with disabilities, which led to inconsistent and often discriminatory practices. Trainers with disabilities from the local communities noted,

“*healthcare workers understood that persons with disabilities are people just like them…and now there is change…when some of us get sick and go to the health facility*,* we are treated well than before.*” (Trainer, with a disability).

Another trainer with disabilities emphasised,

“*Their attitudes and behaviours have changed a lot…healthcare workers attend to persons with disabilities well…if someone with a disability comes last*,* they will attend to you first.*” (Trainer, with a disability).

Three months after the training, healthcare workers described observable changes in their interactions with patients with disabilities. These participants noted that they were able to apply skills and knowledge gained from the training. They described improvements in their practice, routines and decision-making processes for people with disabilities.

“*We used to lose patients to other facilities because of the fear we had treating these patients with disabilities; we had a lot of fear treating them*,* we were afraid coming close to people with disabilities. However*,* we are now more confident because we know that people with disabilities also become sick like we do*,* and sickness affects them the same way. Therefore*,* now we are welcoming to them and there is a very big difference*” (HCW, Female).

Challenges remained, however, in consistently applying new practices due to unsupportive organisational culture, limited resources and limited support from management. Some healthcare workers struggled with integrating the new behaviours into their routine and indicated a need for ongoing support and reinforcement. One participant narrated:

“*Our beds are high… it is a challenge for them to get onto it and we have no solution so we tell them to “try and climb it”. We also need wheelchairs and the condition of our toilets is uncomfortable for patients”* (HCW, Female).

### Level 4: results– organizational benefits

Improvements in patient care and workplace policies were described three months post-training. Participants reported better patient outcomes, in addition to more people with disabilities seeking healthcare services:

“*People with disabilities have increased compared to the initial number we used to get. Based on how they are treated*,* they refer other people with disabilities in the community to this health facility*,* that’s the reason why the number has increased.*” (HCW, female).

Some healthcare workers now use a separate triage process and offer patients with disabilities immediate attention rather than following the standard first-come-first-served rule. This approach has fostered a friendlier, more inclusive environment, with staff intentionally building positive relationships from the first point of contact:

“*We welcome and speak to patients with disabilities when they come to the facility. We also speak to other patients and ask them to give us some time to work with patients with disabilities*,* which wasn’t the case before the training.”* (HCW, Female).

However, mentorship was requested to sustain these improvements and address ongoing challenges. It was also recommended that disability training be included in Continuing Medical Education (CME), sessions that are held monthly as mandated by the Ministry of Health, along with occasional visits to ensure progress towards disability inclusion in health services. Additionally, disability training was recommended to be integrated into pre-service curricula to ensure future healthcare workers are well-prepared to support patients with disabilities.

## Discussion

The disability training programme received positive feedback from participants, who found the practical focus and collaborative learning approach engaging. Key to the success of the training was its delivery by a pair of trainers, a person with a disability and a health worker. Participants showed increased knowledge and skill acquisition, with reported improvements in their interactions with patients with disabilities three months later. However, challenges remained in achieving consistent inclusive health practices, due to unsupportive organisational culture, limited resources, and limited support from management. Ongoing mentorship was requested.

A recent systematic review identified limited qualitative data on healthcare workers’ reported behaviour changes after disability training [7]. Nevertheless, our findings align with other studies that demonstrate the value of disability education in healthcare training through the use of diverse teaching methods and inclusion of the perspectives of people with disabilities [19–21]. For instance, a sexual and reproductive health rights training provided a two-day course for frontline healthcare workers from the Ghana Health Service, increasing their confidence and inclusive behaviours toward patients with disabilities and creating strong demand for further training [21]. Similar to our study, broader system-level changes were found to be necessary for lasting inclusion. As another example, a new module in India’s medical curriculum used storytelling, art, and theatre to help students understand the social challenges faced by people with disabilities. Students gained insight into disability rights, recognised their own misconceptions, and expressed a desire to support inclusion and advocate for change [22]. Ongoing challenges included integrating inclusive practices within resource-limited settings and underscore the need for system-level support to ensure sustained, accessible care across healthcare facilities in India. Thus, while our disability training has shown promise in shifting attitudes and increasing confidence among healthcare providers in rural Uganda, lasting change will require system-level support to integrate inclusive practices and ensure accessible, respectful care for people with disabilities across healthcare settings. Effective implementation of this disability training at scale or in other settings will require the adaption of the training content to align with local practices (e.g. addressing resistance to participatory training approaches) and ongoing evaluation to make the necessary adjustments and ensuring sustained improvements in care.

Strengths of the study include that the training was co-developed using an evidence based and participatory approach and considered the cultural context [23, 24]. People with disabilities are a diverse group with varying needs [3], and the training addressed different types of impairments [25]. The qualitative design of our study allowed for a deep exploration of participants’ experiences and perceptions, providing rich, detailed feedback that revealed the nuanced effects of the disability training om practices. This approach also enabled the identification of emerging themes and areas for improvement through in-depth, context-specific data. Ongoing team discussions throughout data collection and analysis deepened our understanding of the data by applying positionality and reflexivity. However, our study also has limitations. The sample size was localised to Uganda, which may limit the generalisability of findings. The reliance on self-reported data introduces potential biases, as participants may have responded in ways they believed were favourable. The short timeframe post-training may not reflect long-term changes in health service provision. It would also have been useful to include more objective measures of impact, such as patient outcomes or changes in healthcare service provision.

Our findings have important implications for programmes and future research. Specifically, our study suggests that a disability training programme that incorporates the perspectives of both people with disabilities and healthcare workers should emphasise practical, collaborative learning approaches and provide ongoing support to reinforce and sustain changes in health service provision. These findings could inform the adaptation of future training programmes for healthcare workers in Uganda and other resource-limited settings. However, the diverse roles of healthcare workers raise questions about the feasibility of a one-size-fits-all training programme [14]. Policy support is vital to ensure the implementation and support of disability training for healthcare workers, such as mandating inclusion in in-service and pre-service curricula. Future research should explore the effectiveness and scalability of the training across different healthcare cadres and settings to inform broader implementation.

## Conclusion

Our study highlights the positive reception of disability training that includes diverse perspectives and focuses on practical and collaborative learning approaches. There were reported improvements in patient care and workplace practices three months post-training. There is need for ongoing mentorship and institutional support.

## Electronic supplementary material

Below is the link to the electronic supplementary material.

Supplementary Material 1

Supplementary Material 2

## Data Availability

Data is provided in the manuscript and supplementary files.

## Declarations

### Disclaimer

The views and opinions expressed in this article are those of the author(s) and are the product of professional research. It does not necessarily reflect the official policy or position of any affiliated institution, funder, agency, or that of the publisher. The author(s) are responsible for this article’s results, findings, and content. The views expressed are those of the author(s) and not necessarily those of the NIHR or the Department of Health and Social Care.
