# Supplementary material for: Disability training for healthcare workers in Uganda: qualitative findings from the pilot test
Source: BMC Med Educ. 2025 May 23;25:763. doi: 10.1186/s12909-025-07330-4 (PMC12103015; doi:10.1186/s12909-025-07330-4)
Supplement: Supplementary file 1 — Supplementary Material 1 [file 12909_2025_7330_MOESM1_ESM.docx]

**Qualitative Interview guide for Trainers on disability**

**The Missing Billion: Pilot-testing Health worker training approaches to improve access to healthcare for people with disabilities in Uganda**

**Objective:** To gather information to help further design a training of trainers’ programme for healthcare workers about disability

### These questions should be used to guide discussion but do not have to be used in the sequence listed below. The interviewer should follow up on any additional issues that may arise and seem important in relation to the issues above.

### **Introduction**

### Greet them and thank them for their time

### Identify yourself by name and organisation.

### Read out the information sheet. Remind them of confidentiality and anonymity. Check if they have any questions. Remind them that they are free to decline to answer any of the questions or stop the interview at any time.

### Record their consent/assent in the relevant form OR record verbal consent.

### Start recording

### **Notes:** the following details must be recorded in field notes

| Participant Code |  |
| --- | --- |
| Interview date and time |  |
| Interview location or mode (phone, video) |  |
| Interviewer |  |
| Interviewee  (Record caregiver name if they are present) |  |
| Gender |  |
| Age |  |
| General observations (anything which might impact how the interview is conducted) |  |

***Participant background***

1. Can you please tell me about yourself
2. Can you please describe to me or tell me more about your role as [health worker job title or position/role of person with a disability].

**Prompt:** What activities do you do in this role?

1. As we have discussed, we are gathering information to help improve the design of the training of trainers’ programme for healthcare workers about disability that you recently participated in. How has your view of participatory training changed since the training of trainers?

***Participation in training of trainers***

1. Can you describe 2-3 examples of activities in the training that were most helpful to you?

**Prompt:** Kindly share with me why they were useful?

1. Could you describe the ways that you might offer support to health workers as they learn about disability?

**Prompt:** How would you want to do it? What do you think will be easy? What do you think will be difficult?

1. What are some of the lessons that you have learned in the training of trainers about how to teach others about providing holistic consultations to people with disabilities?

**Prompt:** Can you share with me an example of what you might do?

1. What kind of support do you feel you need to effectively train health workers on disability?

Prompt: 1. What do you plan to do differently moving forward?

1. Can you think of any suggestions or feedback you would give to someone who is thinking of training health workers in disability?
2. If we were to deliver the training of trainers again, what could we do differently?
3. Is there anything else that you would like to say about the training of trainers that we have not covered?

***Mentoring on the online learning portal***

1. What will make it easy to mentor health workers online? What will make it difficult?
2. What are some of the things you think will be essential for the mentorship online

**Prompt:** Can you tell me about internet connection and electricity and airtime – How could these be addressed?

1. When would be the most convenient time for you to contribute to an online forum? Can you tell me why?
2. What information about online learning is missing that you would like to know more about?
3. Do you have any questions for me?
